# Longitudinal touchscreen use across early development is associated with faster exogenous and reduced endogenous attention control

**DOI:** 10.1038/s41598-021-81775-7

**Published:** 2021-01-26

**Authors:** Ana Maria Portugal, Rachael Bedford, Celeste H. M. Cheung, Luke Mason, Tim J. Smith

**Affiliations:** 1 Psychological Sciences, Centre for Brain and Cognitive Development, Birkbeck, University of London, London, UK; 2 Center of Neurodevelopmental Disorders (KIND), Division of Neuropsychiatry, Department of Women’s and Children’s Health, Karolinska Institutet, Stockholm, Sweden; 3 Biostatistics and Health Informatics Department, Institute of Psychiatry, Psychology & Neuroscience, King’s College London, London, UK; 4 Department of Psychology, University of Bath, Bath, UK; 5 The Education Endowment Foundation, London, UK

**Keywords:** Psychology, Human behaviour

## Abstract

Childhood *screen time* is associated with both attentional difficulties (for television viewing) and benefits (in action video gamers), but few studies have investigated today’s pervasive *touchscreen* devices (e.g. smartphones and tablets), which combine salient features, interactive content, and accessibility from toddlerhood (a peak period of cognitive development). We tested exogenous and endogenous attention, following forty children who were stable high (HU) or low (LU) touchscreen users from toddlerhood to pre-school. HUs were slower to disengage attention, relative to their *faster* baseline orienting ability. In an infant anti-saccade task, HUs displayed more of a corrective strategy of orienting *faster* to distractors before anticipating the target. Results suggest that long-term high exposure to touchscreen devices is associated with faster exogenous attention and concomitant decreases in endogenous attention control. Future work is required to demonstrate causality, dissociate variants of use, and investigate how attention behaviours found in screen-based contexts translate to real-world settings.

## Introduction

Attention control plays a pivotal role in selecting relevant information from the environment, and is thought to underpin the adaptive control of behaviour early in life^1,2^. This selection results from the interaction between *exogenous* (stimulus-driven and automatic, e.g. looking at a flashed cue) and *endogenous* (goal-driven and voluntary, e.g. inhibition of looking to a distractor) processes^3–5^, and can be studied using saccadic paradigms^6–9^. Although under genetic control, the development of attention is subject to environmental influences^10^, like the visual experience of screen media activity, e.g. television^11^ or computer games^12–14^.

High levels of non-curated television exposure before the age of 2 have been proposed as a risk factor for attention^11,15^ and executive function difficulties^16,17^; with the over activation of exogenous attention by fast-paced TV content^18^, being hypothesized to deplete endogenous attention resources^15,19–22^. However, the evidence to support these associations is often inconsistent, and thorough examinations of the mechanisms for and the directions of the effects are lacking^23^. In contrast, in some studies of adults and older children, action video-games (fast-paced games placing high perceptual and motor demands) have been shown to train attention skills, with video-gamers showing enhanced visual discrimination, processing speed, and endogenous attention^13,24–28^ (however, see^29^ for a review of the counter-evidence). This enhancement is hypothesized to result from a more flexible and efficient allocation of attentional resources^24,26,27^, although the extent to which these effects may also be observed in infants is unknown due to the traditional inaccessibility of action videogames at this age.

Screen media is commonly used as entertainment for children, and with the rapid increase in touchscreen device use (i.e. smartphones and tablets), the media environment of young children has changed, from 28% of 3–4 year-olds using a tablet at home in 2013 to 63% in 2019^30^. Touchscreens share similarities with television, in terms of the salient features that guide attention; and with video-gaming, in the interactivity afforded during video selection and app use. However, research addressing the associations between touchscreen media and cognitive development is limited.

Using the same cohort as the current study, we have shown that, at 18 months and 3.5 years, high touchscreen users (HUs) were faster in exogenous visual search than low users (LUs)—i.e. detecting a red apple amongst blue apples^31^. However, because looking at the most salient item (the red apple) was advantageous, it was not known whether HUs would still display faster exogenous orienting *when such behaviour is in direct conflict with endogenous attention*, e.g. when inhibiting saccades to salient distractors. Preliminary evidence for inhibitory control issues have been reported in pre-schoolers who had high touchscreen app use the year before^32^.

In the current study, we tested whether long-term (from 12–18 months to 3.5 years) use of touchscreens was associated with exogenous and endogenous attention, using two saccadic orienting tasks. These tasks provide objective measures of the interplay between exogenous and endogenous attentional processes and are ideal for investigating attentional control across early development. The *Gap-Overlap* assesses the disengagement and facilitation of attention by measuring the latency of eye movements from a central to a peripheral stimulus^6,8,33^ in three increasing levels of visual competition. In ‘overlap’ trials (highest competition), the two stimuli overlap in time, disrupting automatic saccades and requiring active fixation disengagement, producing longer latencies compared to ‘baseline’ trials (simultaneous peripheral stimulus onset and central stimulus disappearance). In ‘gap’ trials (least competitive condition), a delay between central stimulus disappearance and peripheral onset provides a warning signal, *facilitating* disengagement^34^, producing faster latencies. The *Anti-saccade* indexes inhibition by measuring suppression of automatic saccades to a distractor and execution of an anticipatory saccade in the opposite direction. The adult anti-saccade makes use of instructions^35,36^; in the infant implementation, infants are implicitly trained to look opposite to the distractor by presenting a delayed target stimulus and reinforcing a response to its location with a reward. Over the course of the task, infants learn to inhibit the response to the distractor^37^ to respond quicker to the target and anticipate its appearance. The pro-saccades are thought to reflect exogenous processing, whereas the voluntary anti-saccades reflect endogenous processing^7,9^.

This study aims to test whether HUs’ overt attention shifts (i.e. saccades) (a) were faster than LUs’ under conditions where saliency-driven behaviour is elicited (i.e. exogenous attention, measured by the facilitation index, the latency on the baseline condition, and the proportion and latency of prosaccades) and, critically, (b) differ to LUs when the required shifts conflict with stimulus saliency, requiring endogenous control (the disengagement index and the proportion and latency of anti-saccades).

## Results

### The gap-overlap task

Data from forty children (16 girls) who were either High (26 HUs) or Low (14 LUs) users across visits (long-term users) were included in the analysis. Groups did not differ on the number of valid trials (see Supplementary Table S6 online).

A GEE model including usage group (HU, LU) and visit (12 months, 18 months and 3.5 years) as predictors of *disengagement* showed a significant main effect of group (*p* = 0.047, see Table 1): LUs showed a smaller disengagement index (mean = 101 ms) compared to HUs (mean = 130 ms). There were no significant effects of visit nor interactions.

**Table 1:** Summary of GEE Model Effects including long-term user group (high and low users) and visit (12 months, 18 months, and 3.5 years) as predictors of the Gap-Overlap Task outcome measures.

	Wald χ^2^ (df), p- value
**Disengagement**	
Visit	4.51 (2), p = 0.105
*Group*	**3.95 (1), p = 0.047**
Visit * *group*	0.33 (2), p = 0.848
**Facilitation**	
Visit	**15.20 (2), p = 0.001**
*Group*	1.13 (1), p = 0.288
Visit * *group*	0.40 (2), p = 0.817
**Follow-up model on baseline latency**	
Visit	**7.62 (2), p = 0.022**
*Group*	**4.99 (1), p = 0.026**
Visit * *group*	3.06 (2), p = 0.216
**Follow-up model on overlap latency**	
Visit	**18.22 (2), p < 0.001**
*Group*	0.01 (1), p = 0.919
Visit * *group*	2.89 (2), p = 0.235

The analysis included 14 LUs and 26 HUs.

Significant results are in bold.

For *facilitation*, there was a significant main effect of visit (*p* = 0.001, see Table 1). Bonferroni corrected pairwise comparisons showed a significantly smaller facilitation index at 12 months (mean = − 69 ms) relative to 3.5 years (mean = − 22 ms, *p* < 0.001), but no differences with 18 months (mean = − 52 ms, *p* = 0.318 and *p* = 0.073 respectively). There was no group main or interaction effects.

**Figure 1:**
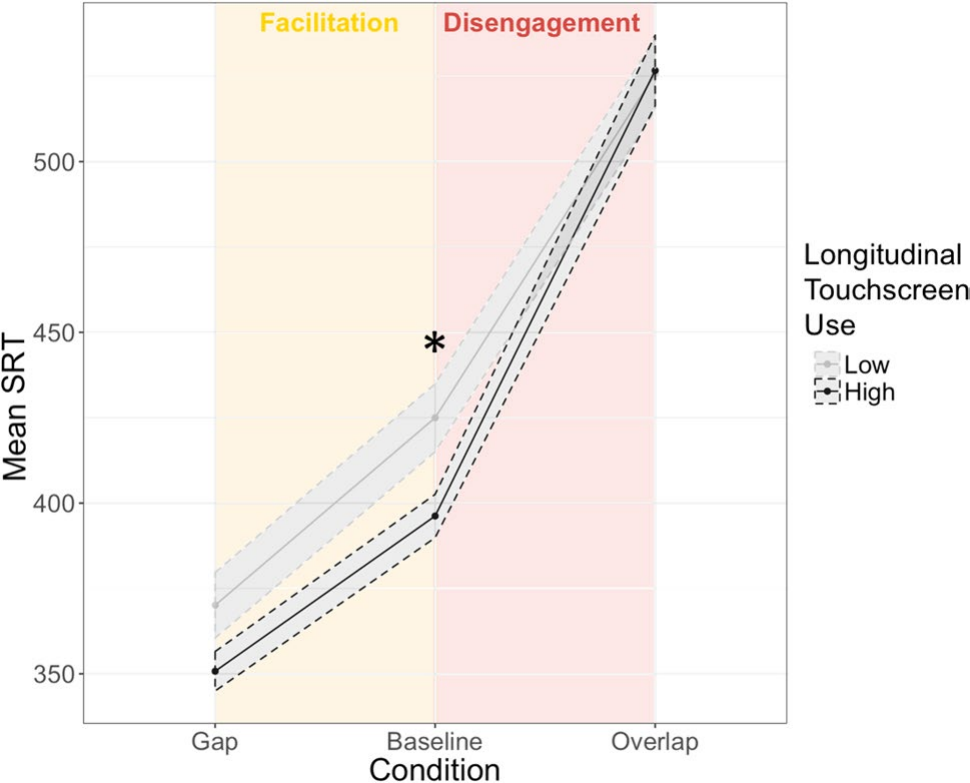
Mean Saccadic Reaction Time (ms) for each touchscreen use group (N = 40) as a function of trial condition in the Gap-Overlap Task. Measures are aggregated across the three longitudinal visits. Shaded areas represent standard error of the mean. *p < 0.05.

To follow-up on the group effect on disengagement, separate GEEs models were run for saccadic latencies in the Baseline and Overlap condition. Latency in the *baseline condition* was significantly associated with user group (*p* = 0.026, see Table 1) with HUs showing faster baseline latencies (mean = 396 ms) compared with LUs (mean = 425 ms)—see Fig. 1; there was also a main effect of visit (*p* = 0.022), with faster latencies at 3.5 years (mean = 392 ms) relative to 12 months (mean = 425 ms, *p* = 0.019), but not 18 months (mean = 412 ms, *p* = 0.119 and *p* = 0.596 respectively). The interaction was not significant. For latency in the *overlap condition*, a GEE model showed a main effect of visit (*p* < 0.001, see Table 1), with faster latencies at 3.5 years (mean = 487 ms) compared with 12 months (mean = 548 ms, *p* < 0.001) and 18 months (mean = 537 ms, *p* = 0.002), but no difference between 12 and 18 months (*p* = 0.959). There was no main or interaction effect of group.

There was no significant main effects of sex (*p* > 0.2) or of average Background TV (*p* > 0.2) on the outcome variables.

In summary, results show that the disengagement index was higher for HUs, suggesting reduced endogenous attention. However, HUs were faster than LUs only in the baseline condition, suggesting faster exogenous attention in this group.

### The anti-saccade task

#### Proportion of saccadic behaviour

Data from 38 children (16 girls) who were either High (24 HUs) or Low (14 LUs) users across visits were included in the analysis. Groups did not differ on the number of valid trials (see Supplementary Table S6 online).

A GEE model with group, visit, and task half (first, second) as predictors of the *proportion of anti-saccades* showed a main effect of half (*p* < 0.001, see Table 2), with a higher proportion in the second half of the task (mean = 0.46), compared with the first half (mean = 0.12); suggesting all participants were learning the task. There was also a main effect of visit (*p* = 0.005). Bonferroni corrected pairwise comparisons showed that at 18 months (mean = 0.40) children were doing more anti-saccades than at 12 months (mean = 0.25, *p* = 0.016), however, at 3.5 years (mean = 0.22), they were doing less anti-saccades than at 18 months (*p* = 0.004), with no significant difference between 3.5 years and 12 months (*p* > 0.99). There was no main effect of touchscreen user group, and no significant interaction effects.

**Table 2:** Summary of GEE Model Effects including long-term user group (high and low users) and visit (12 months, 18 months, and 3.5 years), and task half (first, second) as predictors of the Anti-saccade Task outcome measures.

	Wald χ^2^ (df), p-value
**% Anti-saccades**	
Half	**125.02 (1), p < 0.001**
Visit	**10.80 (2), p = 0.005**
*Group*	0.14 (1), p = 0.706
Half * *group*	1.30 (1), p = 0.254
Visit * *group*	2.00 (2), p = 0.369
Half * visit	5.46 (2), p = 0.065
Half * visit * *group*	0.82 (2), p = 0.661
**% Pro-saccades**	
Half	**230.34 (1), p < 0.001**
Visit	**10.03 (2), p = 0.007**
*Group*	< 0.01 (1), p = 0.966
Half * *group*	0.03 (1), p = 0.871
Visit * *group*	1.49 (2), p = 0.476
Half * visit	**10.56 (2), p = 0.005**
Half * visit * *group*	0.13 (2), p = 0.938
**% Corrective looks**	
Half	**5.01 (1), p = 0.025**
Visit	**14.29 (2), p = 0.001**
*Group*	3.26 (1), p = 0.071
Half * *group*	**9.35 (1), p = 0.002**
Visit * *group*	1.37 (2), p = 0.505
Half * visit	**6.52 (2), p = 0.038**
Half * visit * *group*	1.08 (2), p = 0.583
**Latency to distractor (pro-saccade)**	
Half	< 0.01 (1), p = 0.993
Visit	**17.70 (2), p < 0.001**
*Group*	**4.55 (1), p = 0.033**
Half * *group*	1.75 (1), p = 0.186
Visit * *group*	4.96 (2), p = 0.084
Half * visit	**9.06 (2), p = 0.011**
Half * visit * *group*	1.69 (2), p = 0.430
**Latency to target location (anti-saccade)**	
Half	**9.28 (1), p = 0.002**
Visit	**33.69 (2), p < 0.001**
*Group*	0.94 (1), p = 0.334
Half * *group*	1.71 (1), p = 0.191
Visit * *group*	0.26 (2), p = 0.878
Half * visit	4.04 (2), p = 0.133
Half * visit * *group*	0.79 (2), p = 0.673

The analysis included 14 LUs and 24 HUs.

Significant results are in bold.

For the *proportion of pro-saccades* (i.e. looks to the distractor not followed by an anticipatory look to the target location) there was again an effect of half (*p* < 0.001, the proportion decreased from the first, mean = 0.71, to the second half, mean = 0.32; see Table 2); and an effect of visit (*p* = 0.007), with more pro-saccades at 12 months (mean = 0.61) than 18 months (mean = 0.44, *p* = 0.007) and 3.5 years (mean = 0.51, *p* = 0.051), while at 18 months and 3.5 years the proportion did not differ (*p* > 0.99). There was also an interaction between half and visit (*p* = 0.005); follow-up models split by half showed that at 18 months and 3.5 years babies started with a similar proportion of pro-saccades (which was lower than at 12 months), but by the second half 18-month-olds had a lower proportion of pro-saccades compared to 12-month-olds and 3.5-year-olds—i.e. children at 3.5 years do not seem to reduce the proportion of pro-saccades across the task as much as the toddlers (see means in Supplementary Table S7 online). There was no effect of group, or other interactions.

**Figure 2:**
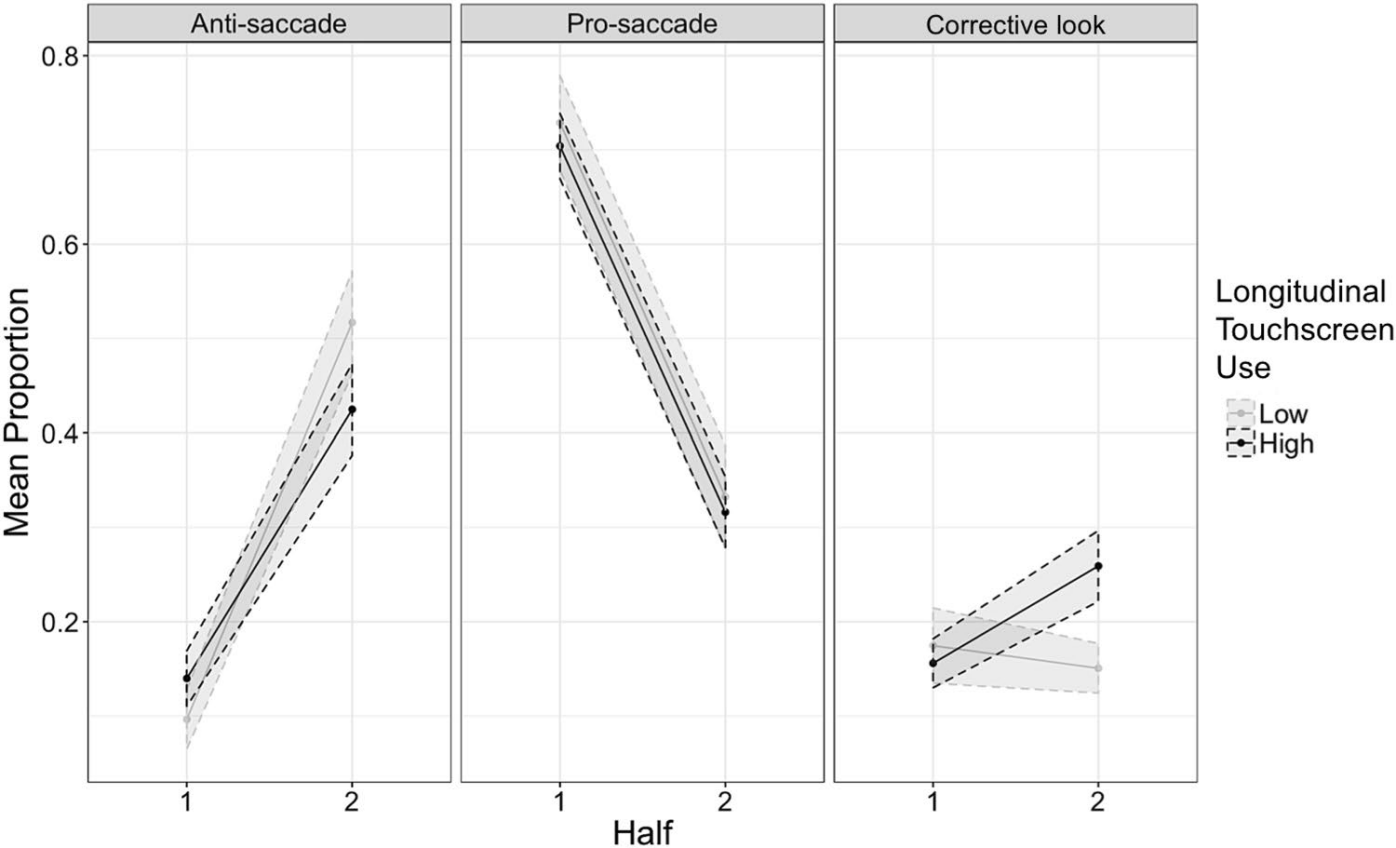
Mean proportion for each longitudinal touchscreen use group (N = 38) as a function of Task Half and look behaviour in the Anti-Saccade Task. Measures are aggregated across the three longitudinal visits. Shaded areas represent standard error of the mean.

For the *proportion of corrective looks* (looks to the distractor followed by an anticipatory look to the target location) there was a main effect of half (*p* = 0.025, the proportion of corrective looks increased from the first, mean = 0.16, to the second half, mean = 0.22; see Table 2), and a main visit effect (*p* = 0.001), with children doing more corrective looks at 3.5 years (mean = 0.27) than at 12- (mean = 0.14, *p* = 0.008) and 18-months (mean = 0.16, *p* = 0.001), while 12 and 18 months did not differ (*p* > 0.99). There was also an interaction between half and visit (*p* = 0.038); follow-up models for each half showed that visit differences were only evident in the first half of the task (*p* = 0.008). There was no main effect of group, but there was a significant interaction effect of half and group (*p* = 0.002). Follow-up models showed that groups differed in their corrective looks in the second half of the task (*p* = 0.017)—see Fig. 2. There were no other interactions.

In summary, results suggest that participant behaviour adapted appropriately to the infant anti-saccade task as indexed by an increase in anti-saccades (endogenous attention) during the task. Performance seems to be optimal at 18 months with the highest proportion of anti-saccades in the second half at this age; whereas performance at 3.5 years is similar to performance at 12 months. However, in the first block of the task at 12 months there were more pro-saccades (exogenous attention), while at 3.5 years there were more corrective looks (failing to inhibit a pro-saccade but still anticipating the target). In terms of usage group, while no differences in proportion of anti-saccades (endogenous) or pro-saccades (exogenous) was found, HUs showed more of this corrective behaviour in the second half of the task.

#### Latencies to distractor and target

A GEE model for the *latency to the distractor during a pro-saccade* showed a main effect of visit (*p* < 0.001, see Table 2 above), with children being faster at 3.5 years (mean = 436 ms) than at 12 (mean = 526 ms, *p* = 0.005) and 18 months (mean = 548 ms, *p* = 0.017), while no difference between 12 and 18 months was found (*p* > 0.99). This model also showed a main effect of group (p = 0.033): LUs were slower (mean = 535 ms) than HUs (mean = 480 ms). There was no main effect of half, but there was an interaction between half and visit (*p* = 0.011); follow-up models run for each half showed that the visit effect was only evident in the first half (*p* < 0.001).

For the *latency to saccade to the target location during an anti-saccade* there was a main effect of half (*p* = 0.002, see Table 2), with latencies decreasing from the first (mean = 702 ms) to the second half (mean = 665 ms). The model also showed an effect of visit (*p* < 0.001): at 18 months (mean = 619 ms) anti-saccades were faster than at 12 months (mean = 719 ms, *p* < 0.001), but at 3.5 years (mean = 716) they were slower than at 18 months (*p* = 0.010), with a similar level of performance compared with 12 months (*p* > 0.99). There was no main effect of group, and no interactions. See Supplementary Table S8 online for means of latency measures. In summary, results suggest that, in line with the proportion analysis, performance was better at 18 months; with faster latencies to anti-saccade at this age. In terms of pro-saccades, latencies were faster at 3.5 years in the first half of the task; and HUs were faster than LUs throughout the task (suggesting faster exogenous attention).

See all GEE results with covariates in Supplementary Table S9 online. There was a main effect of sex only for the latency to saccade to the target location during an anti-saccade (*p* = 0.023), with girls being faster to anti-saccade than boys; results remained similar to the ones presented above when controlling for it. There was a significant main effect of Background TV on the proportion of anti-saccades (*p* = 0.002), pro-saccades (*p* = 0.018) and corrective looks (*p* = 0.001), with higher Background TV associated with less anti-saccades, more pro-saccades and more corrective looks; higher Background TV was also associated with faster latencies to saccade to the target location during an anti-saccade (*p* = 0.002). When running the analysis with Background TV as a covariate all main and interaction effects reported above remained significant, apart from the main effect of half on the proportion of corrective looks, which became marginally significant (*p* = 0.076).

### Concurrent time-varying group analysis

To assess if the results above were specific to long-term touchscreen use (which indexes concurrent and past consistent usage), all analyses were repeated with the full-sample and the time-varying concurrent touchscreen use group as predictor. Results are reported in Supplementary Note S10 online.

In the Gap-overlap Task (n = 53), visit effects remained similar; in terms of touchscreen usage group (HU, LU), the effect did not reach significance for the *disengagement index* (*p* = 0.123) but remained significant for the *baseline condition* latency (*p* = 0.016).

In the infant Anti-saccade Task (n = 51), effects remained mostly similar. The two exceptions were that the interaction of half and visit was significant for *proportion of anti-saccades* (*p* = 0.041; at 18 months children were doing more anti-saccades than at 12 months and 3.5 years in the second half); and that the interaction of task half and visit did not reach significance for *corrective looks* (*p* = 0.141). The usage group effect on the *latency to the distractor during a pro-saccade* remained significant between concurrent groups (*p* = 0.025), as did the interaction of task half and usage for proportion of corrective looks (*p* = 0.007).

## Discussion

Long-term touchscreen use was associated with differences in the speed and control of attention allocation over the visual scene across two tasks, with HUs showing faster exogenous attention (the baseline and pro-saccades latencies) and concomitant endogenous attention differences (longer disengagement index). Concurrent use was associated with faster exogenous attention.

On the Gap-overlap task, long-term HUs were slower to disengage attention. However, this was due to them being faster when shifting attention on a no-competition condition, rather than being slow on the overlap-competition condition. One well-documented change after playing video-games is faster reaction times^25,28,38^; however, in this study, a general increase in processing speed was not found. Rather, considering the speed advantages HUs presented in the baseline, HUs took more time than expected to disengage attention in the overlap condition. The saliency bias (faster baseline latencies) was found for concurrent and long-term HUs, which supports the idea that exposure to touchscreens, which provide experience with salient and contingent content, may lead to a greater attentional bias to exogenous salient stimuli, in line with our previous finding of faster pop-out search in HUs^31^. Although significant group effects on the facilitation index (which was thought to index exogenous attention) and on the gap condition (which also indexes a shift to a salient stimuli, *p* = 0.158, see Supplementary Table S11 online) were not found, given that facilitation is a subtraction of gap and baseline latencies and the later differed between groups, it is reasonable to say that HUs either tended to also be faster in the gap condition or tended to have a weaker facilitation effect. It is possible that the complex processes that underlie facilitation (e.g. phasic alerting^10^) might be implicated in high users of touchscreens, but this hypothesis cannot be addressed with the studies presented.

On the Anti-saccade task, HUs produced more corrective looks, still anticipating the target. HUs were also faster to look to the salient distractor, again supporting our previous finding^31^, which may have triggered the corrective behaviour (see below). While anti-saccade performance increased from 12 to 18 months, at 3.5 years, children produced fewer and slower anti-saccades while being faster to shift to the distractor and producing more corrective looks (see Supplementary Figure S11 online for a visualization of age differences in performance). It is possible that the target onset delay (1000 ms after distractor offset at all visits, necessary to ensure measures could be compared across visits) was too generous to enforce automatic saccade inhibitions at 3.5 years, allowing children with faster orienting to opt for an overselective behaviour, i.e. look to distractor and anticipate the target. The similar direction of effects between visit and touchscreen usage tentatively suggests that HUs’ faster exogenous attention enables them to opt for this corrective, overselective behaviour already at 12 and 18 months. Corrective saccades, which were also found in other studies of the anti-saccade with young children^7^, could suggest that participants learnt the constraints of the task and were able to adapt to it given their exogenous orienting speed. Alternatively, this corrective behaviour could be seen as a failure to inhibit a pro-saccade while still anticipating the reward. The finding that at 3.5 years the latency to anti-saccade is slower than at 18 months suggests that participants struggled to anti-saccade at this age. It is important to highlight that while proportion and latency of anti-saccades did not statistically differ between usage groups, descriptively, they tended towards reduced and slower inhibitory control also in HUs (i.e., less and slower anti-saccades). Dissociating these two hypotheses (i.e. does corrective behaviour reflect an overselective adaptation or a failure to inhibit attention?) is crucial to understand the implications to attention and executive control of the differences found, and future studies should look at trial-by-trial performance to understand the different learning strategies used at different ages and usage levels. In terms of pro-saccades (thought to index exogenous attention), while no difference between the groups was found, it is important to note this exogenous behaviour is also captured by the corrective looks found to be different between groups (these behaviours were constrained to be mutually exclusive, such that a pro-saccade that occurred during a corrective look did not count for the final proportion of pro-saccades).

In sum, these findings indicate that the visual attention of young HU children may be more exogenously driven than that of LUs—replicating our previous finding of faster pop-out visual search^31^—but also demonstrating that the association is both with concurrent and long-term touchscreen use, and extending it to tasks in which such behaviour is not always advantageous to performance. On the other hand, endogenous control appears reduced in long-term HUs only, with slower disengagement of attention (relative to their baseline). These endogenous differences may be driven by a long-term exogenous speed advantage, which allows them to opt for a different attentional strategy. It may be that HUs use their faster orienting to compensate for endogenous differences, or alternatively, that their strategy to give priority to automatic exogenous processing displaces opportunities for learning endogenous control over the critical first few years of neurodevelopment, as already suggested in relation to television viewing^15,19–22^.

The use of a longitudinal design and objective lab-based attention measures in the current study provided a detailed profile of attention performance associated with using a touchscreen early in life. However, this approach has clear limitations. First, given that the findings are based on associations, it is equally possible that children who are already biased towards salient content (and have relatively reduced endogenous attention control) are predisposed towards touchscreens, or that a sensitivity to saliency (and concomitant difficulties) is caused by using a touchscreen. While direction and causality remain to be investigated, the concurrent associations found in this and our previous study (and the changed group memberships over the years) tentatively suggest that the saliency bias seen in HUs is not due to trait-level predispositions and that this attentional profile was acquired through the experience with touchscreens. Second, given the screen-based tasks administration, it is unknown if the behaviours found are screen-specific, and future investigations should replicate these in “real-world” settings where saliency appears in the form of distraction and executive processes are required more actively to control behaviour in a goal-driven way^39,40^, such as solving a puzzle in a busy living room, or concentrating in a classroom.

Another limitation is that the assignment of touchscreen use group was based on a parent-report question, which may be subject to reporter bias and under-estimation^41^. However, response to this question was strongly correlated with the cumulative duration of daily touchscreen use reported in at least one media diary kept by their parents during a day prior to each visit (see correlations statistics in “Methods”—“Touchscreen use” section and in Supplementary Table S3 online; see Vandewater and Lee^42^ for discussion of the suitability of such diaries when objective measurement is not possible). Further, the rank order of objectively-measured individual differences in screen time is captured by self/parent-report, such as the median split global estimate used in this study^43^. However, future studies should attempt to use objective tracking of the duration, context and content of touchscreen exposure, in order to understand how variants of use are associated with attention control. As far as we know, the findings of faster exogenous attention have not been documented before in relation to more conventional media (i.e. television and video-gaming), which suggests a potentially unique role of these devices for the developing mind. While at our age range the use of a touchscreen is predominantly to watch videos, with increasing age the type of usage also seems to change from more passive to more active use^44^. A lack of interaction effects between age and usage in our results could tentatively suggest the effects found are not dependent on the type of touchscreen use (e.g. watching videos versus playing games) and may be specific to the experience afforded by the touchscreen platform. It is, however, crucial to follow-up these findings by studying or manipulating the context and content of such experience to try and pinpoint the specific characteristics of the platform that are associated with these attentional patterns.

In conclusion, the results presented suggest that long-term exposure to touchscreen media is associated with faster exogenous orienting, and concomitant reduced endogenous attention control (slower disengagement of attention). If replicated in larger-scale future studies this finding could have important implications for the development of digital media content and evidenced-based screen-time policies.

## Methods

### Participants and study design

Fifty-six infants were recruited between October 2015 and March 2016, through the Birkbeck and Goldsmith’s Babylab databases and communication and social media. Three participants were later excluded from the study—one withdrew consent after the first visit, and the other two received a later diagnosis of genetic or neurological conditions. Families visited the Babylab and children took part in a battery of experimental measures (Supplementary Table S1 online), including the saccadic control tasks described below, as part of three longitudinal visits at 12 months (N = 53, 23 girls, M = 376 days, SD = 20), 18 months (N = 49, 22 girls, M = 540 days, SD = 21) and 3.5 years (N = 46, 23 girls, M = 1256, SD = 16). Full sample details are reported in Supplementary Table S2 online. One child was born prematurely at 32 weeks, and one child occasionally suffers from Reflex Anoxic Seizures—as both were able to fully perform the tasks their data were retained in the analysis. The study was approved by the Birkbeck Psychological Sciences ethics board and conducted according to the British Psychological Society Code of Ethics and Conduct. Parents provided written informed consent at each visit.

### Measures

#### Touchscreen use

Parents assessed their child’s touchscreen use in hours and minutes before each visit, through a question embedded in an online survey: ‘On a typical day, how long does your child spend using a touchscreen device (tablet, smartphone or touchscreen laptop)?’^45^. Infants were initially recruited and assigned to a user group based on the median for average daily touchscreen use in 12- to 13-month-olds from a previous online survey sample^45^, ≥ 10 min/day “high users” (HU), < 10 min/day “low users” (LU). At subsequent visits, the median was calculated within the sample: 15 min/day at 18 months and 3.5 years (Table 3). This parent-reported duration of use was significantly associated with total touchscreen exposure in 24-h media diaries (12 months: r_s_ = 0.49; 18 months: r_s_ = 0.59; 3.5 years: r_s_ = 0.62; see Supplementary Table S3 online). At recruitment, groups were matched on background covariates—see Supplementary Table S2 online for detailed descriptive statistics of concurrent usage groups.

**Table 3:** Parent-reported touchscreen use (minutes/day) details for the TABLET sample and concurrent usage groups (LU = low user, HU = high user) split by visit.

	12-months (min/day)	18-months (min/day)	3.5-years (min/day)
12-months (min/day)	–	0.78**	0.31*
Correlations between visits			
18-months (min/day)	–	–	0.33*
**Concurrent usage**			
All sample			
Median cut-off	10	15	15
Sample mean (SD)	25.58 (54.94)	29.14 (62.28)	37.93 (62.92)
LU			
N	21	23	19
Mean (SD)	0.52 (1.25)	2.09 (3.23)	3.16 (4.15)
HU			
N	32	26	27
Mean (SD)	42.03 (66.01)	53.08 (78.54)	62.41 (73.03)

*p < .05, **p < .001 for the Spearman’s rho correlation.

For some children, touchscreen usage was not consistent throughout the study and their group membership changed between visits (as evidenced by the moderate to high correlations for duration of use between visits, see Table 3). For this reason, only participants who had stable usage over time (n = 40), and hence their touchscreen use across visits could index long-term exposure, were considered in the main analysis; however, concurrent time-varying group analysis (i.e. cross-sectional) using the full sample (n = 53) can be seen in the Supplementary File online. To be considered as a long-term user, a child’s user group at either of the first ‘toddler’ visits (12 or 18 months) needed to match the usage group at the 3.5 year ‘preschool’ visit (i.e. if at 12 months a child was a LU and at 3.5 years he/she was also a LU, then he/she would be considered a long-term LU; if at 12 and 18 months a child was a HU but at 3.5 years he/she was a LU, then he/she would be considered an unstable user and hence dropped from this analysis). If children missed the last visit, they were included in a group if their usage was consistent on the other time points (this happened for 5 children). See Supplementary Table S4 online for the possible group permutations and outcome group classification. In total, 14 children were classified as LUs, 26 were HUs and 13 children were dropped from analysis because their usage could not be described across age points.

#### Background covariates

Table 4 presents detailed descriptive statistics for each long-term usage group, including touchscreen media use duration and background measures: sex, age at each visit, general development level at 12 months (assessed by the Mullen Scales of Early Learning^46^), average Background TV Viewing (min/day assessed through the question: “On a typical day, how long is a TV switched on in your home?”), and Mothers’ education. Given that sex and average background TV were significantly different across groups, these were tested as covariates in follow-up analysis, with any that had a significant main effect on the outcome retained and reported in the Supplementary File online.

**Table 4:** Descriptive and frequency statistics for key background variables by long-term touchscreen media user group.

	Long-term low users	Long-term high users	Between-groups comparison
N	14	26	
**Touchscreen use**			
Average min/day	3 (5)	54 (78)	**p = 0.003**
**Sex**			
Girls	9 (64%)	7 (27%)	**p = 0.021**
Boys	5 (36%)	19 (73%)	
**Mother’s education**			
School-leaving, college	0	3 (11%)	n.s. (p = 0.177)
University, postgrad	14 (100%)	22 (85%)	
Missing/N/A	0	1 (4%)	
**Age (days)**			
At 12-months	378 (16)	375 (21)	n.s. (p = 0.687)
At 18-months	542 (18)	540 (16)	n.s. (p = 0.748)
At 3.5-years	1253 (13)	1257 (20)	n.s. (p = 0.499)
**Background TV**			
At 12-months^a^	127 (178)	230 (174)	n.s. (p = 0.084)
Average^a^	118 (169)	236 (171)	**p = 0.042**
**MSEL standard score**			
At 12-months	111 (11)	108 (11)	n.s. (p = 0.372)

For continuous numerical variables data is presented as mean (standard deviation) and difference between user groups (high and low users) was tested with an independent samples t-test. For categorical variables data is presented as N (proportion) and difference between user groups (high and low users) was tested with a Pearson Chi-Square.

Significant results are in bold

^a^One value that exceed 3 standard deviations from the mean were trimmed (i.e. changed to be one more than the non-trimmed highest value).

#### Lab-measures of attention control

Attention control performance was measured in the lab on two gaze-contingent paradigms. Participants’ eye coordinates were recorded at 120 Hz using a Tobii TX300 eye-tracker (Tobii Technology, Stockholm, Sweden), MATLAB, and the Tobii Analytics SDK on a MacBook Pro. Stimuli were presented on a 23″ widescreen monitor (16:9, 1920X1080 pixels) with stereo speakers via custom scripts using PsychToolbox (version 3.0.12) while the child was seated on their parent’s lap approximately 60 cm distance. The session was monitored and recorded with a web camera located above the screen with the ScreenFlow (Telestream Inc., version 9.0) screen-casting software. Participants’ gaze was calibrated using a child appropriate 5-points procedure^47^ before each task. After calibration, stimulus presentation ran automatically (pacing of the trials and the timing of stimuli presentation was dependent on child’s gaze) and continued until the end unless children became overly fussy.

**Figure 3:**
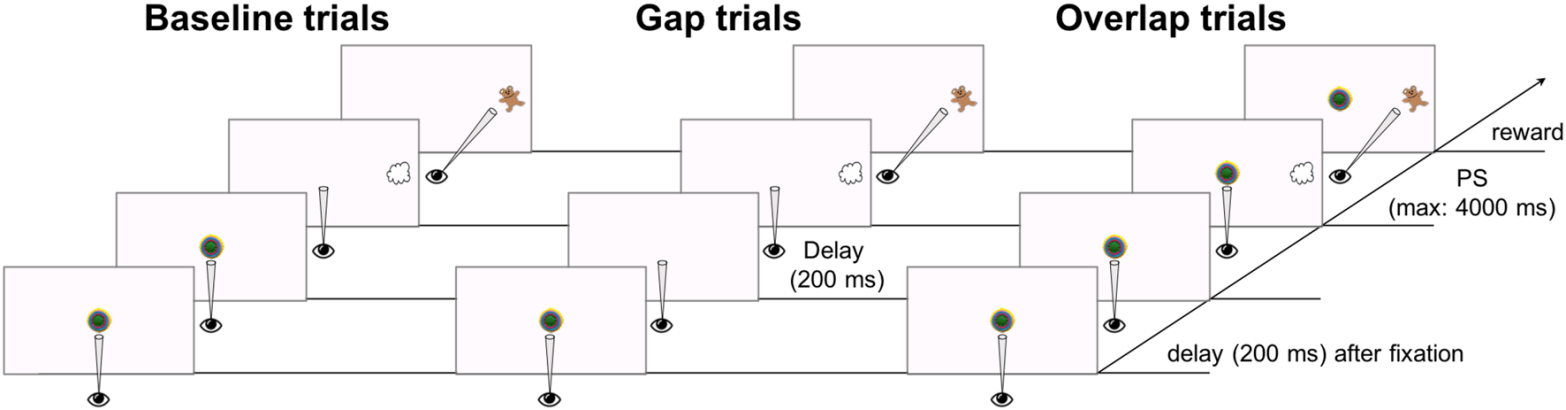
Stimulus sequence for experimental trials in the Gap-Overlap Task. Stimuli drawn to scale. Every trial started with the central stimulus onset and were followed by the presentation of the peripheral stimuli (PS).

The Gap-Overlap task was presented first, across seven blocks of 12 trials interleaved with free-viewing of dynamic and static scenes. All trials began with a centrally presented animation (CS, subtending 6.5° × 6.4°) to attract the child to the centre of the screen—see Fig. 3. Once the child fixated the CS and after a delay of 200 ms, a peripheral target (PS, a cloud subtending 6° × 6°) was presented randomly to either left or right side of the screen, at the eccentricity of 18.5°. For 25% of trials, the PS was presented either on top or bottom of CS to avoid anticipation, ‘vertical trials’, but these were not included in the analysis. When the child looked to the PS, or after 4 s elapsed, a novel animated stimuli (reward) replaced it and the trial ended. In the Overlap condition, the PS appeared while the CS remained displayed so that the two stimuli overlapped until the end of the trial; in the Baseline condition, the CS disappeared and the PS appeared simultaneously; in the Gap condition, the CS disappeared and was followed by a gap of 200 ms before the PS appeared.

The conditions were presented pseudo-randomly within block: 40% of these trials were Overlap trials, 30% were Baseline trials and another 30% were Gap trials. A maximum of 70 trials (ignoring the vertical trials) was presented. The central stimulus and the background colour changed every block.

Saccadic latencies (ms) were defined as the time from the PS presentation onset to the first look to the PS and were extracted offline. All trials were automatically validated based on gaze quality flags and latency duration (see processing details in the OSF archived file at https://osf.io/p5ahq/). Only valid trials were considered when averaging latency for each condition. Disengagement was then calculated by subtracting the baseline latency from the overlap latency, and facilitation by subtracting the baseline latency from the gap latency.

**Figure 4:**
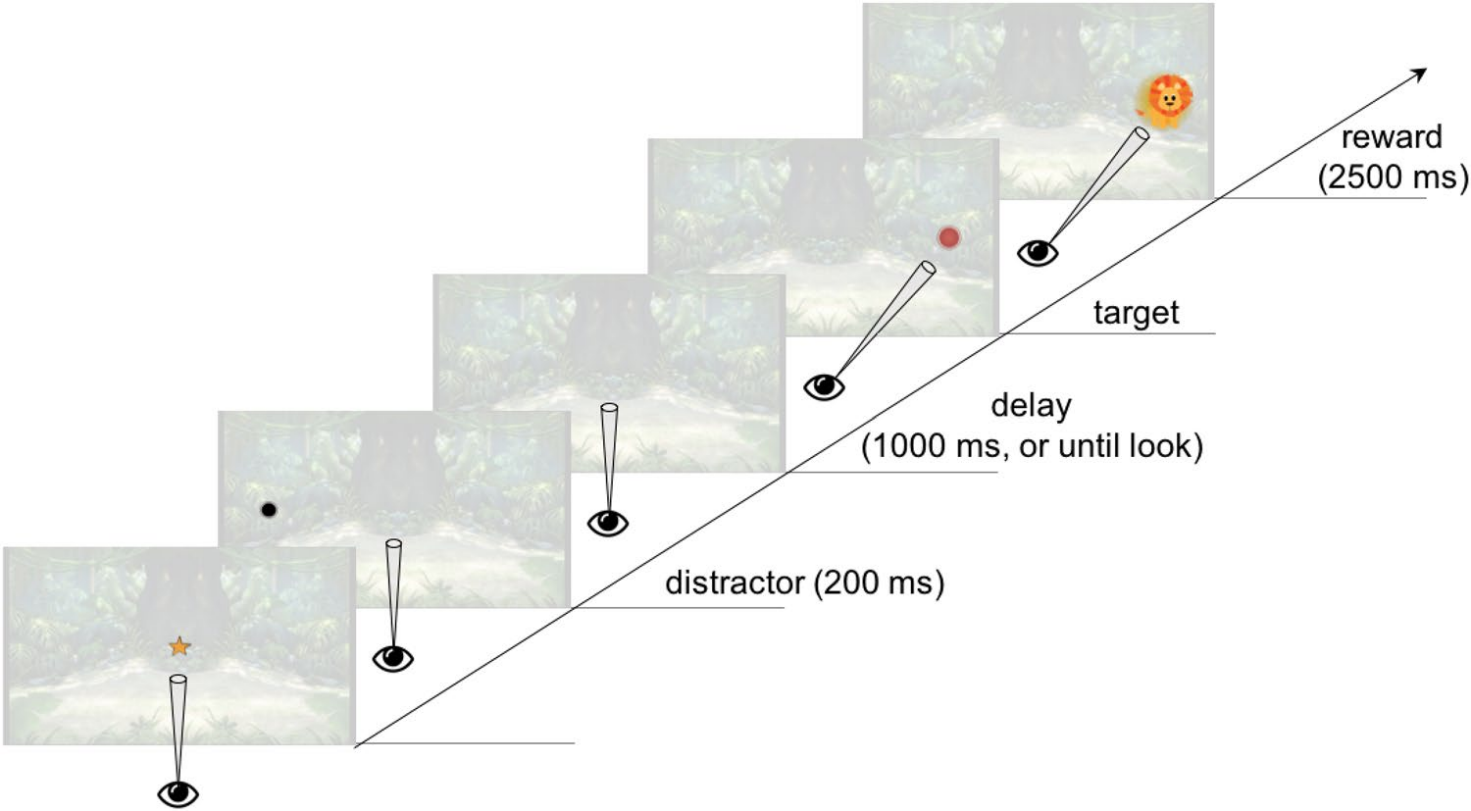
Stimulus sequence for experimental trials in the Anti-Saccade Task. Stimuli drawn to scale. Every trial started with the central fixation stimulus onset.

The Anti-saccade task was presented in a second block of tasks and all trials started with the presentation of a central animation (a star, subtending 3° × 3°) to attract the child to the centre of the screen—see Fig. 4. When the participant looked to this central stimulus, a distractor stimulus (a black circle, subtending 3° × 3° with 17° to the right or left of the screen) appeared for 200 ms. Only 1000 ms after the distractor disappeared a target stimulus (a red circle, subtending 4° × 4° with 17° eccentricity) was presented on the opposite side. When the child looked to the target an attractive animation of an animal with sound replaced it and the trial ended. If the participant looked at the target side before its presentation, the animation started immediately. Within participant, the Distractor and Target did not change sides across trials but side was balanced between groups. The task was presented in one continuous series of trials, consisting of 26 (at the 12-month visit) or 15 (at 18 months and 3.5 years) trials.

Location of looks and reaction times to stimuli were measured offline. In each trial, it was determined (1) whether the participant looked at the distractor and (2) whether he/she looked at the target location before (or shortly after, up to 100 ms post-target onset, as per other studies using anti-saccade paradigms in infants^7^, and adults^35^) the onset of the target (= anticipatory look). All trials were automatically validated based on gaze quality flags (see processing details in the OSF archived file at https://osf.io/p5ahq/). If during a trial the child did not look to the distractor nor the target location before target appearance the trial was excluded on the basis that the child failed to orient to the distractor. Only valid trials were considered for further computation of measures.

The first 15 trials were segmented in two, first half ‘first 7 trials’ and second half ‘remaining 8 trials’. The proportion of looks towards the distractor not followed by an anticipatory look (= pro-saccades); of looks towards the distractor followed by an anticipatory look (= corrective saccades); and of anticipatory looks in the absence of a look to the distractor (= true anti-saccades, where inhibition of pro-saccades, as well as the production of contralateral saccades is required) were calculated for each half (categories were mutually exclusive in a trial), as well as the average latency to the cue during a pro-saccade and to the target location during an anti-saccade.

### Analytic approach

The data analysis plan for the 3.5-year visit was pre-registered on the Open Science Framework^48^ which can be accessed at https://osf.io/fxu7y. In a deviation from this plan, touchscreen effects were tested using long-term exposure and linear Generalised Estimating Equation (GEE) models with identity link and unstructured correlation matrix. GEE is an ideal method for analysing longitudinal data and commonly used in experimental repeated-measures data similar to this study^31,49^ as it takes into account the within subject change over time, while allowing us to include individuals who had missing data points (but see Supplementary Note S5 online for the pre-registered ANOVA analysis results). Missing data points occurred due to unavailability to come to the lab, technical problems, excessive fussiness, or low number of valid trials on the task (less than 5). For Gap-Overlap, two GEE models were run, with disengagement and facilitation indexes as outcome variables and visit and long-term usage group as predictors. For the Anti-saccade Task, separate GEE models for the proportion of anti-, corrective-, and pro-saccades, and the latencies to distractor and target were run with half, visit, and the usage group as predictors. Main effects models were run first and then 2-way and 3-way interaction effects were added in sequential steps. When age effects were found, they were followed up by Bonferroni corrected pairwise comparisons to assess differences between each age level.

## Supplementary Information

Supplementary Information.

## Data Availability

The datasets generated during and/or analysed during the current study can be accessed in OSF at https://osf.io/s374m/?view_only=6edda5bbc4b248cebe377dd807b65fb6.

## Code availability

Code used to process and extract the measures from the eye movements data can be accessed in OSF at https://osf.io/9guzq/?view_only=26461456d02a428a9aaffef3b7e9a871.
