# Supplementary material for: Longitudinal touchscreen use across early development is associated with faster exogenous and reduced endogenous attention control
Source: Sci Rep. 2021 Jan 26;11:2205. doi: 10.1038/s41598-021-81775-7 (PMC7838402; doi:10.1038/s41598-021-81775-7)

**Supplementary Information**

Longitudinal touchscreen use across early development is associated with faster exogenous and reduced endogenous attention control

Ana Maria Portugal^1,2^, Rachael Bedford^3^, Celeste H. M. Cheung^1,4^, Luke Mason^1^, Tim J. Smith^1^*

^1^ Centre for Brain and Cognitive Development, Birkbeck, University of London

^2^ Center of Neurodevelopmental Disorders (KIND), Division of Neuropsychiatry, Department of Women’s and Children’s Health, Karolinska Institutet, Stockholm, Sweden

^3^ Biostatistics and Health Informatics Department, Institute of Psychiatry, Psychology & Neuroscience, King’s College London

^4^ The Education Endowment Foundation, London, UK.

* Corresponding author: Tim J. Smith, Psychological Sciences, Birkbeck, University of London; [tj.smith@bbk.ac.uk](mailto:tj.smith@bbk.ac.uk)

Supplementary Table S1. Task battery for the lab-sample at each time point.

|  | 12 months | 18 months | 3.5 years |
| --- | --- | --- | --- |
| Parent-report surveys completed online |  |  |  |
| Basic demographic variables including Mother’s and Father’s Education, Date of Birth, Gender, and Gestation | *🗸* | - | - |
| Media use questionnaire | 🗸 | 🗸 | 🗸 |
| Media diary | 🗸 | 🗸 | 🗸 |
| Very-short-IBQ/very-short-ECBQ/short-CBQ | 🗸 | 🗸 | 🗸 |
| Sleep | 🗸 | 🗸 | 🗸 |
| Parent-report surveys completed in the lab |  |  |  |
| Vineland-II Adaptive Behavior Scales | - | - | 🗸 |
| CBCL | - | 🗸 | 🗸 |
| SDQ | - | - | 🗸 |
| Eye-tracking based measures |  |  |  |
| Gap-overlap Task (Block 1) | 🗸 | 🗸 | 🗸 |
| Free-viewing of dynamic and static scenes (Block 1) | 🗸 | 🗸 | 🗸 |
| Memory task (Block 1) | 🗸 | 🗸 | - |
| Anti-saccade Task (Block 2) | 🗸 | 🗸 | 🗸 |
| Visual Search Task (Block 2) | - | 🗸 | 🗸 |
| Inhibition task (Block 2) | 🗸 | - | - |
| Sequence learning task (Block 2) | - | 🗸 | - |
| Lab-based measures |  |  |  |
| Mullen Scales of Early Learning | 🗸 | 🗸 | (VR Scale) |
| Parent-child play | 🗸 | 🗸 | 🗸 |
| EEG | 🗸 | 🗸 | - |
| Executive Function battery | - | - | 🗸 |
| Visual function battery | - | - | 🗸 |
| Lab-based physiological measures |  |  |  |
| Activity | 🗸 | 🗸 | 🗸 |
| Other: Heart Rate, GSR, Temperature | 🗸 | - | 🗸 |

IBQ = Infant Behavior Questionnaire; ECBQ = Early Childhood Behavior Questionnaire; CBQ = Childhood Behavior Questionnaire; CBCL = Child Behavior Checklist; SDQ = Strengths and Difficulties Questionnaire; VR = Visual Reception

Supplementary Table S2. Descriptive and frequency statistics for the lab-sample and touchscreen user groups at each age visit. For continuous numerical variables data is presented as Mean (Standard Deviation) and difference between user groups was tested with an independent samples T-Test. For categorical variables data is presented as N (Proportion) and difference between user groups was tested with a Pearson’s Chi-Square.

| At 12 months |  |  |  |  |
| --- | --- | --- | --- | --- |
|  | Sample | Low users  (<10 min/day) | High users  (≥10 min/day) | Difference between groups |
| N | **53** | **21** | **32** |  |
| Touchscreen use (min/day) | **26 (55)** | **0.5 (1)** | **42 (66)** | ***p = .*001** |
| Sex |  |  |  |  |
| Girls | 23 (43%) | 12 | 11 | *n.s. (.102)* |
| Boys | 30 (57%) | 9 | 21 |  |
| Mother’s Education |  |  |  |  |
| School leaving or college | 4 (7%) | 1 | 3 | *n.s. (.565)* |
| University or postgrad | 48 (91%) | 19 | 29 |  |
| Missing, N/A | 1 (2%) | 1 |  |  |
| Age (days) | 376 (20) | 374 (19) | 378 (20) | *n.s. (.426)* |
| Background TV (min/day) * | 180 (171) | 151 (170) | 199 (172) | *n.s (.323)* |
| Mullen Standard Score | 108 (11) | 110 (10) | 107 (12) | *n.s. (.446)* |

* One value that exceed 3 standard deviations from the mean was trimmed (i.e. changed to be one more than the non-trimmed highest value)

| At 18 months |  |  |  |  |
| --- | --- | --- | --- | --- |
|  | Sample | Low users  (<15 min/day) | High users  (≥15 min/day) | Difference between groups |
| **N** | **49** | **23** | **26** |  |
| **Touchscreen use (min/day)** | **29 (62)** | **2 (3)** | **53 (79)** | ***p = .*003** |
| **Sex** |  |  |  |  |
| **Girls** | 22 (45%) | 13 | 9 | n.s. (.124) |
| **Boys** | 27 (55%) | 10 | 17 |  |
| **Mother’s Education** |  |  |  |  |
| **School leaving or college** | 3 (6%) | 2 | 1 | n.s. (.502) |
| **University or postgrad** | 45 (92%) | 21 | 24 |  |
| **Missing, N/A** | 1 (2%) |  | 1 |  |
| **Age (days)** | 540 (21) | 536 (19) | 544 (22) | n.s. (.165) |
| **Background TV (mind/day)** | **146 (124)** | **105 (112)** | **182 (124)** | ***p = .*030** |
| **Mullen Standard Score** | 111 (18) | 113 (18) | 108 (18) | n.s. (.364) |

| At 3.5 years |  |  |  |  |
| --- | --- | --- | --- | --- |
|  | Sample | Low users  (<15 min/day) | High users  (≥15 min/day) | Difference between groups |
| **N** | **46** | **19** | **27** |  |
| **Touchscreen use (min/day)** | **38 (63)** | **3 (4)** | **62 (73)** | ***p* < 0.001** |
| **Sex** |  |  |  |  |
| **Girls** | 23 (50%) | 12 | 11 | n.s. (.134) |
| **Boys** | 23 (50%) | 7 | 16 |  |
| **Mother’s Education** |  |  |  |  |
| **School leaving or college** | 3 (7%) | 0 | 3 | n.s. (.125) |
| **University or postgrad** | 42 (91%) | 19 | 23 |  |
| **Missing, N/A** | 1 (2%) |  | 1 |  |
| **Age (days)** | 1256 (16) | 1257 (14) | 1256 (18) | n.s. (.781) |
| **Background TV (min/day)** | **169 (140)** | **112 (147)** | **208 (123)** | ***p = .*022** |

Supplementary Table S3. Parent-reported touchscreen use (minutes/day) and parent-reported Background TV (minutes/day) cross-sectional (i.e. concurrent) correlations with media-diary estimates of total touchscreen time, total TV viewing time, and total TV time split by adult-directed and child-directed TV programmes viewing. Spearman’s rho correlations were computed with trimmed usage values (values that exceed 3 standard deviations from the mean were changed to be one more than the non-trimmed highest value). Significant correlations are in bold.

|  | *Questionnaire* | Media-diary estimates (hours/day) | | | |
| --- | --- | --- | --- | --- | --- |
|  | *Background TV* | *Total Touchscreen* | *Total TV* | *Adult TV* | *Child TV* |
| At 12 months |  |  |  |  |  |
| *Touchscreen use, r_s_*  *p*  *N* | 0.17  0.227  53 | **0.49**  **<0.001**  **53** | 0.25  0.075  53 | 0.20  0.160  53 | 0.14  0.313  53 |
| *Background TV, r_s_*  *p*  *N* |  | 0.20  0.161  53 | **0.64**  **<0.001**  **53** | **0.36**  **0.008**  **53** | **0.46**  **0.001**  **53** |
| At 18 months |  |  |  |  |  |
| *Touchscreen use, r_s_*  *p*  *N* | **0.38**  **0.009**  **48** | **0.59**  **<0.001**  **49** | **0.31**  **0.031**  **49** | 0.25  0.080  49 | 0.24  0.100  49 |
| *Background TV, r_s_*  *p*  *N* |  | **0.32**  **0.026**  **48** | **0.63**  **<0.001**  **48** | **0.44**  **0.002**  **48** | **0.49**  **<0.001**  **48** |
| At 3.5 years |  |  |  |  |  |
| *Touchscreen use, r_s_*  *p*  *N* | **0.40**  **0.007**  **45** | **0.62**  **<0.001**  **44** | **0.30**  **0.049**  **44** | -0.03  0.83  44 | 0.27  0.079  44 |
| *Background TV, r_s_*  *p*  *N* |  | **0.40**  **0.009**  **43** | **0.66**  **<0.001**  **43** | 0.19  0.229  43 | **0.48**  **<.0001**  **43** |

**Supplementary Table S4.** Group permutations and outcomes for recoding of the longitudinal stable group

To recode touchscreen usage group so it could index children’s cumulative touchscreen use across visits, the below scheme was used. To be considered for a stable usage group the child’s group at infancy visits would need to match the usage group at the pre-school visit. If children missed visits, they were still included in a group if their usage was consistent on the other time points.

| INFANCY | | | |  | PRE-SCHOOL | OUTCOME CUMULATIVE GROUP |  |
| --- | --- | --- | --- | --- | --- | --- | --- |
| 12 mo |  | **18 mo** |  | | **3.5 y** |  | N |
| LU | **⇒** | LU | **⇒ ⇒** | | LU | **Low User** | 12 |
| HU | **⇒** | LU | **⇒ ⇒** | | LU |  | 2 |
| HU | **⇒** | HU | **⇒ ⇒** | | HU | **High User** | 17 |
| LU | **⇒** | HU | **⇒ ⇒** | | HU |  | 1 |
| HU | **⇒** | LU | **⇒ ⇒** | | HU |  | 3 |
| HU | **⇒** | HU |  | | *Miss* |  | 2 |
| HU |  | *Miss* |  | | *Miss* |  | 3 |
|  |  |  |  | |  |  |  |
| LU | **⇒** | LU | **⇒ ⇒** | | HU | **Dropped from analysis** | 6 |
| HU | **⇒** | HU | **⇒ ⇒** | | LU |  | 5 |
| LU | **⇒** | HU |  | | *Miss* |  | 1 |
| LU |  | *Miss* |  | | HU |  | 1 |
|  |  |  |  | |  | **Total** | 53 |

**Supplementary Note S5.** Results of the pre-registered analysis

The Gap-Overlap task

On the pre-registered 3.5 years longitudinal assessment plan effects in disengagement and facilitation in the Gap-overlap Task at 3.5 years were proposed to be tested in a repeated-measures ANOVA with effect type (disengagement, facilitation) as within subject factors and concurrent usage group (high, low) as between subject factor. The results of this pre-registered analysis were not similar to the ones reported with the GEE, see Table below, due to the different analysis and independent variable used (main analysis tested cumulative touchscreen use, and the pre-registered analysis was planned to test concurrent touchscreen use). At 3.5 years, there was a main effect of the type of effect measured in the task – this is predicted given that the disengagement and facilitation are different in their direction (disengagement has a positive value, because overlap SRT > baseline SRT, whereas facilitation has a negative value, because gap SRT > baseline SRT). There was no other main effects or interactions in the pre-registered repeated-measures ANOVA. However, descriptively, high users tended to have a higher disengagement effect, as it was reported in the GEE – see Tables below.

Summary statistics of the pre-registered repeated-measures ANOVA with type (disengagement, facilitation) as within subject factors and touchscreen usage group (high, low) as between factor, in the Gap-overlap Task at 3.5 years. The sample included 19 LUs and 26 HUs.

|  | **Visit at 3.5 years** |
| --- | --- |
| **Pre-registered repeated-measures ANOVA: magnitude of effect** | F (df_IV_, df_error_), p value |
| **Effect type (disengagement, facilitation)** | **110.91 (1,43), p < .001** |
| ***Group*** | 2.52 (1,43), p = .120 |
| **Effect type**Group*** | 0.10 (1,43), p = .756 |
|  |  |

Mean and Standard Deviation of disengagement and facilitation effects in the Gap-Overlap Task, for each touchscreen use group at 3.5 years.

| Visit at 3.5 years | Concurrent Touchscreen use group | Mean | SD |
| --- | --- | --- | --- |
| Disengagement | LU | 96 | 77 |
|  | HU | 118 | 83 |
| Facilitation | LU | -44 | 58 |
|  | HU | -14 | 60 |

The Anti-saccade task

Effects in the Anti-saccade Task were meant to be examined using a t-test with proportion of anticipatory looks (i.e. any trial where the child looked at the target location before the onset of the target, or shortly after, up to 100 ms post-target onset) as the dependent variable and concurrent usage group (high, low) as the grouping variable. Results from this analysis did not show any significant difference between high and low users at 3.5 years in terms of proportion of anticipatory looks to the target location, t(42) = 0.44, p = .659 – see Table below. These anticipatory looks measure includes corrective looks and anti-saccades, and thus the analysis presented in the main text, dissociating the various looking behaviours in the task, is a more suitable approach.

Mean and Standard Deviation of proportion of anticipatory looks in the Anti-saccade, for touchscreen use group at 3.5 years, including 18 LUs and 26 HUs.

| Visit at 3.5 years | Concurrent Touchscreen use group | Mean Proportion | SD |
| --- | --- | --- | --- |
| Anticipatory looks | LU | 0.47 | 0.27 |
|  | HU | 0.44 | 0.21 |

**Supplementary Table S6.** Number of participants and number of valid trials, represented in terms of Mean (Standard Deviation), by task, age visit and user group. Difference between user groups was assessed on an independent samples T-Test.

|  | Low users | High users | Between-groups comparison |
| --- | --- | --- | --- |
| Gap-overlap Task |  |  |  |
| N, Mean number of trials (SD) |  |  |  |
| At 12-months | 14, 47 (15) | 26, 44 (15) | n.s. (p = .528) |
| At 18-months | 14, 50 (9) | 23, 49 (14) | n.s. (p = .787) |
| At 3.5-years | 14, 44 (9) | 19, 50 (9) | n.s. (p = .092) |
| Antisaccade Task |  |  |  |
| N, Mean number of trials (SD) |  |  |  |
| At 12-months | 13, 11 (3) | 18, 11 (3) | n.s. (p = .834) |
| At 18-months | 12, 12 (3) | 22, 11 (3) | n.s. (p = .327) |
| At 3.5-years | 13, 12 (2) | 19, 11 (3) | n.s. (p = .260) |

**Supplementary Table S7.** Mean and Standard Deviation of Proportion of look behaviour in the Anti-saccade Task

Mean and Standard Deviation of proportion of each saccadic behaviour in the Anti-Saccade Task for each half of the task and age point.

| Type | Age | Half | Mean % | SD |
| --- | --- | --- | --- | --- |
| Anti-saccade | 12 months | 1 | 0.11 | 0.21 |
|  |  | 2 | 0.39 | 0.34 |
|  | 18 months | 1 | 0.19 | 0.25 |
|  |  | 2 | 0.61 | 0.34 |
|  | 3.5 years | 1 | 0.07 | 0.15 |
|  |  | 2 | 0.36 | 0.35 |
| Pro-saccade | 12 months | 1 | 0.82 | 0.21 |
|  |  | 2 | 0.39 | 0.31 |
|  | 18 months | 1 | 0.68 | 0.32 |
|  |  | 2 | 0.20 | 0.27 |
|  | 3.5 years | 1 | 0.65 | 0.28 |
|  |  | 2 | 0.38 | 0.31 |
| Corrective looks | 12 months | 1 | 0.07 | 0.11 |
|  |  | 2 | 0.21 | 0.29 |
|  | 18 months | 1 | 0.14 | 0.23 |
|  |  | 2 | 0.18 | 0.19 |
|  | 3.5 years | 1 | 0.28 | 0.24 |
|  |  | 2 | 0.25 | 0.27 |

**Supplementary Table S8.** Mean and Standard Deviation of Saccadic Reaction Time in the Anti-saccade Task

Mean and Standard Deviation of Saccadic Reaction Time in milliseconds to the distractor (during a pro-saccade) and to the target (during an anti-saccade) in the Anti-Saccade Task, for each Longitudinal touchscreen use group.

| Direction | Longitudinal touchscreen use group | Mean SRT | SD |
| --- | --- | --- | --- |
| To distractor  (during a pro-saccade) | LU | 535 | 154 |
|  | HU | 480 | 129 |
| To Target location  (during an anti-saccade) | LU | 642 | 248 |
|  | HU | 700 | 244 |

Mean and Standard Deviation of Saccadic Reaction Time in milliseconds to the distractor (during a pro-saccade) and to the target (during an anti-saccade) in the Anti-Saccade Task, for each Longitudinal touchscreen use group at each age point.

| Age | Direction | Longitudinal touchscreen use group | Mean SRT | SD |
| --- | --- | --- | --- | --- |
| 12 months | To distractor  (during a pro-saccade) | HU | 518 | 106 |
|  |  | LU | 538 | 104 |
|  | To Target location  (during an anti-saccade) | HU | 749 | 281 |
|  |  | LU | 677 | 272 |
| 18 months | To distractor  (during a pro-saccade) | HU | 522 | 149 |
|  |  | LU | 600 | 197 |
|  | To Target location  (during an anti-saccade) | HU | 661 | 232 |
|  |  | LU | 551 | 186 |
| 3.5 years | To distractor  (during a pro-saccade) | HU | 409 | 96 |
|  |  | LU | 481 | 149 |
|  | To Target location  (during an anti-saccade) | HU | 709 | 217 |
|  |  | LU | 722 | 271 |

**Supplementary Table S9.** Results covarying for Background TV and gender

Summary of GEE models including half, group, and age as predictors of proportion and latency of saccadic behaviour in the Anti-saccade Task when adding background TV or sex as covariates. Only models where the covariate had a significant main effect are shown. The analysis included 14 LUs and 24 HUs.

|  | *Background TV as covariate* Wald χ^2^ (df), p value | *Sex as covariate*  Wald χ^2^ (df), p value |  |
| --- | --- | --- | --- |
| % Anti-saccades | | | |
| Half | **121.16 (1), p < .001** |  |  |
| Visit | **10.50 (2), p = .005** |  |  |
| *Group* | 0.33 (1), p = .567 |  |  |
| *Covariate* | **9.67 (1), p = .002** |  |  |
| Half**Group* | 1.66 (1), p = .198 |  |  |
| Visit**Group* | 2.05 (2), p = .358 |  |  |
| Half*Visit | 5.51 (2), p = .064 |  |  |
| Half*Visit**Group* | 0.81 (2), p = .668 |  |  |
|  | | | |
| % Pro-saccades | | | |
| Half | **256.78 (1), p < .001** |  |  |
| Visit | **8.55 (2), p = .014** |  |  |
| *Group* | 0.25 (1), p = .619 |  |  |
| *Covariate* | **5.62 (1), p = .018** |  |  |
| Half**Group* | 0.04 (1), p = .849 |  |  |
| Visit**Group* | 1.89 (2), p = .388 |  |  |
| Half*Visit | **10.83 (2), p = .004** |  |  |
| Half*Visit**Group* | 0.17 (2), p = .917 |  |  |
|  | | | |
| % Corrective looks | | | |
| Half | 3.16 (1), p = .076 |  |  |
| Visit | **9.73 (2), p = .008** |  |  |
| *Group* | 0.37 (1), p = .546 |  |  |
| *Covariate* | **12.00 (1), p = .001** |  |  |
| Half**Group* | **5.60 (1), p = .018** |  |  |
| Visit **Group* | 0.28 (2), p = .869 |  |  |
| Half* Visit | **6.55 (2), p = .038** |  |  |
| Half* Visit **Group* | 1.41 (2), p = .493 |  |  |
|  | | | |
| SRT to target location (anti-saccade) | | | |
| Half | **4.23 (1), p = .040** | **11.146 (1), p = .001** |  |
| Visit | **22.40 (2), p < .001** | **51.728 (2), p < .001** |  |
| *Group* | 1.22 (1), p = .269 | 0.213 (1), p = .645 |  |
| *Covariate* | **9.29 (1), p = .002** | **5.15 (1), p = .023** |  |
| Half**Group* | 0.97 (1), p = .324 | 1.88 (1), p = .171 |  |
| Visit **Group* | 0.39 (2), p = .824 | 0.21 (2), p = .900 |  |
| Half* Visit | 4.79 (2), p = .091 | 3.71 (2), p = .157 |  |
| Half* Visit **Group* | 0.27 (2), p = .872 | 0.902 (2), p = .637 |  |

**Supplementary Note S10.** GEE models with concurrent touchscreen usage group as a time-varying predictor

For the **Gap-Overlap task** (N = 53), GEE models were run including concurrent usage group (HU, LU) and visit (12, 18 months and 3.5 years) as predictors of disengagement, facilitation, and latencies on each condition of the task. There were no significant main effects of Background TV (unmatched covariate between groups) on any of the outcome variables (*p* > 0.2).

|  | ***Concurrent usage group model*** |
| --- | --- |
|  | Wald χ^2^ (df), p value |
| **Disengagement** | |
| Visit | 2.80 (2), p = .247 |
| *Group* | 2.37 (1), p = .123 |
| Visit **Group* | 0.26 (2), p = .877 |
|  | |
| **Facilitation** | |
| **Visit** | **24.90 (2), p < .001** |
| *Group* | 3.23 (1), p = .072 |
| Visit **Group* | 6.34 (2), p = .042 |
|  |  |
| **Facilitation at 12 months** | |
| *Group* | 2.78 (1), p = .095 |
|  | |
| **Facilitation at 18 months** | |
| *Group* | 0.94 (1), p = .332 |
|  |  |
| **Facilitation at 3.5 years** | |
| *Group* | 3.16 (1), p = .076 |
|  | |
| **SRT Baseline** | |
| **Visit** | **13.24 (2), p = .001** |
| ***Group*** | **5.83 (1), p = .016** |
| Visit **Group* | 2.91 (2), p = .233 |
|  | |
| **SRT Overlap** | |
| **Visit** | **17.33 (2), p < .001** |
| *Group* | <0.01 (1), p = .980 |
| Visit **Group* | 0.82 (2), p = .663 |
|  |  |

For the **Anti-saccade task** (N = 51), GEE models were run including concurrent usage group (HU, LU), visit (12, 18 months and 3.5 years), and task half (first, second) as predictors of proportion of anti-saccades, pro-saccades, corrective looks, latency to saccade to the distractor (during a pro-saccade) and latency to saccade to the target side in the absence of a prosaccade (i.e. during an anti-saccade). There was a significant main effect of Background TV on the proportion of anti-saccades (*p = .*015), with higher concurrent Background TV associated with less anti-saccades. The analysis with Background TV as a covariate showed a main effect of age, a main effect of half, and an interaction effect between age and half, as seen in the main model. There were no other significant main effects of concurrent Background TV on any of the outcome variables.

|  | *Concurrent usage group model* Wald χ^2^ (df), p value |
| --- | --- |
| % Anti-saccades | |
| Visit | **8.22 (2), p = .016** |
| Half | **146.88 (1), p < .001** |
| *Group* | 0.10 (1), p = .749 |
| Visit *Half | **6.38 (2), p = .041** |
| Visit **Group* | 0.89 (2), p = .641 |
| Half **Group* | 0.62 (1), p = .430 |
| Visit *Half **Group* | 3.77 (2), p = .152 |
|  | |
| % Pro-saccades | |
| Visit | **6.02 (2), p = .049** |
| Half | **260.71 (1), p < .001** |
| *Group* | 0.02 (1), p = .882 |
| Visit *Half | **6.73 (2), p = .035** |
| Visit **Group* | 0.87 (2), p = .648 |
| Half **Group* | 0.092 (1), p = .762 |
| Visit *Half **Group* | 1.43 (2), p = .490 |
|  | |
| % Corrective looks | |
| Visit | **8.14 (2), p = .017** |
| Half | **8.19 (1), p = .004** |
| *Group* | 0.06 (1), p = .808 |
| Visit *Half | 3.92 (2), p = .141 |
| Visit **Group* | 1.12 (2), p = .572 |
| Half **Group* | **7.41 (1), p = .007** |
| Visit *Half **Group* | 4.92 (2), p = .085 |
|  | |
| SRT to distractor (pro-saccade) | |
| Visit | **15.35 (2), p < .001** |
| Half | 0.54 (1), p = .464 |
| *Group* | **5.00 (1), p = .025** |
| Visit *Half | **6.62 (2), p = .036** |
| Visit **Group* | 3.22 (2), p = .200 |
| Half **Group* | 3.48 (1), p = .062 |
| Visit *Half **Group* | 0.95 (2), p = .622 |
|  | |
| SRT to target location (anti-saccade) | |
| Visit | **34.85 (2), p < .001** |
| Half | **7.36 (1), p = .007** |
| *Group* | 3.31 (1), p = .069 |
| Visit *Half | 0.69 (2), p = .709 |
| Visit **Group* | 2.90 (2), p = .235 |
| Half **Group* | 2.91 (1), p = .088 |
| Visit *Half **Group* | 3.05 (2), p = .218 |

**Supplementary Table S11.** GEE Model effects on the Gap SRT of the Gap-Overlap Task

GEE Model effects including longitudinal user group and visit as predictors of the Gap condition SRT. The analysis included 14 LUs and 26 HUs.

|  | Wald χ^2^ (df), p value |
| --- | --- |
| **Follow-up model on Gap SRT** | |
| Visit | 0.53 (2), p = .767 |
| *Group* | 2.00 (1), p = .158 |
| Visit **Group* | 1.70 (2), p = .428 |

**Supplementary Figure S11.** Mean Proportion of Look behaviours for Longitudinal touchscreen user group (lines; N=38) as a function of Task Half, visit (12 months, 18 months, and 3.5 years) and type of look behaviour in the Anti-Saccade Task. Shaded areas represent standard error of the mean.


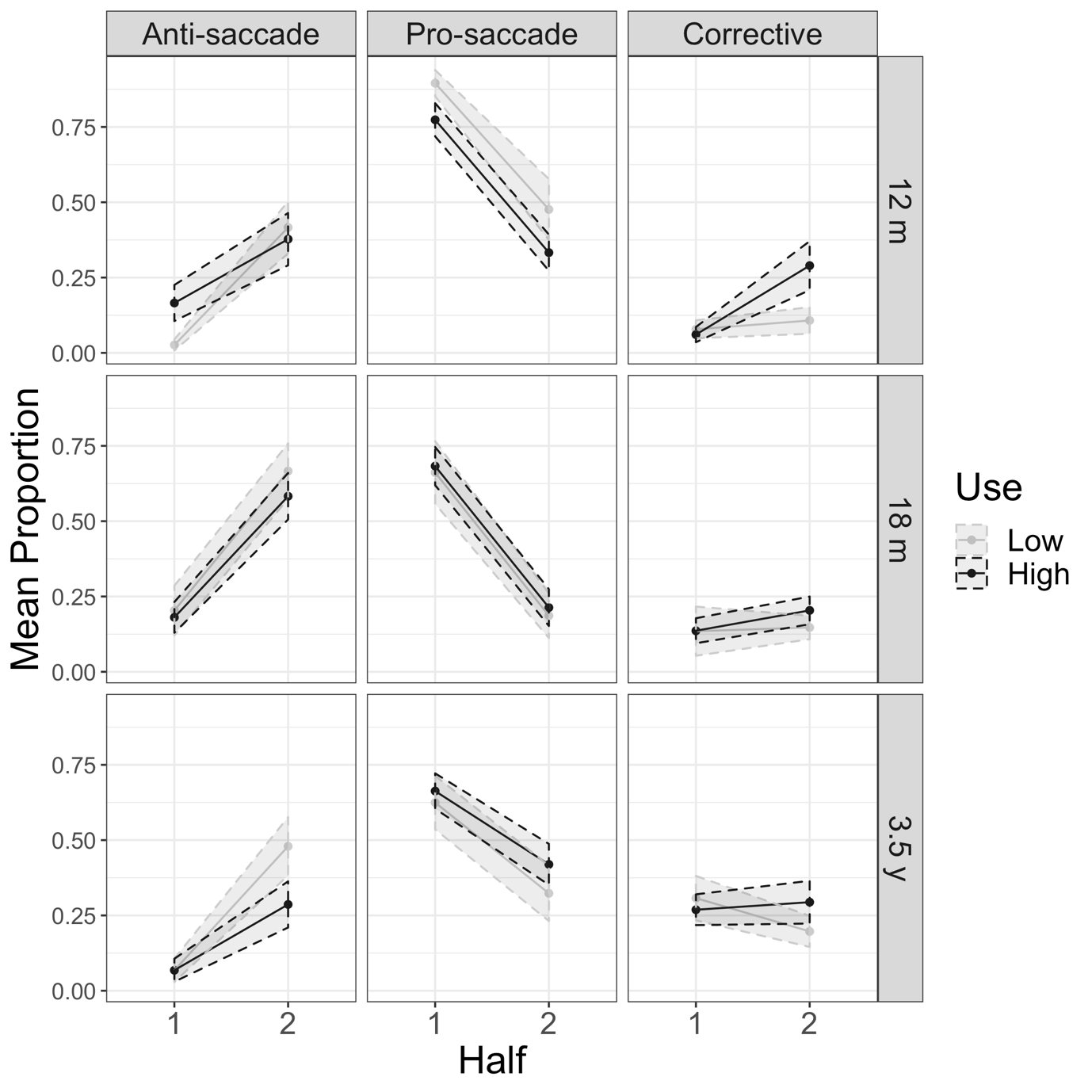

Supplement: Supplementary file 1 — Supplementary Information. [file 41598_2021_81775_MOESM1_ESM.docx]
